# Supplementary material for: Subjective data, objective data and the role of bias in predictive modelling: Lessons from a dispositional learning analytics application
Source: PLoS One. 2020 Jun 12;15(6):e0233977. doi: 10.1371/journal.pone.0233977 (PMC7292385; doi:10.1371/journal.pone.0233977)
Supplement: S1 Appendix — (PDF) [file pone.0233977.s001.pdf]

Appendix A: instruments of self-report surveys.

All surveys address student learning in the Quantitative Methods (QM) course. Therefore, all items mentions as a context: ‘In my learning for the QM course, …’. For reference to the article, the equations and the figures, all variables are indicated with their full name, acronym and number.

A.1 Achievement emotions

The Control-Value Theory of Achievement Emotions (CVTAE; [23-25]) postulates that emotions that arise in learning activities differ in valence, focus, and activation. Emotional valence can be positive (enjoyment) or negative (anxiety, hopelessness, boredom). CVTAE describes the emotions experienced about an achievement activity (e.g. boredom experienced while preparing homework) or outcome (e.g. anxiety towards performing at an exam). The activation component describes emotions as activating (i.e. anxiety leading to action) versus deactivating (i.e. hopelessness leading to disengagement). For this study, we made a selection of four scales measuring learning emotions, found to be most strongly related to course performance, from the Achievement Emotions Questionnaire [44], next to Academic Control as the common antecedent of all learning emotions:

- LEnjoyment (LJO,1): positive, activating learning emotion; sample item: I enjoy acquiring new knowledge.
- LAnxiety (LAX, 2): negative, activating learning emotion; sample item: I get tense and nervous while studying.
- LBoredom (LBO, 3): neutral, deactivating learning emotion; sample item: The material bores me to death.

- LHopelessness (LHL, 4): negative, deactivating learning emotion; sample item: I feel hopeless when I think about studying.
- Academic Control (ASC, 5): antecedent of all learning emotions; sample item: I have a great deal of control over my academic performance.

A.2 Epistemic emotions

While achievement emotions, described in the previous section, arise from doing learning activities, like doing homework, epistemic emotions are related to cognitive aspects of the task itself [24, 25]. Prototypical epistemic emotions are curiosity and confusion. In this study, epistemic emotions were measured with the Epistemic Emotion Scales (EES; [45]). That instrument includes the scales:

- Surprise (6): neutral epistemic emotion; sample item: surprised.
- Curiosity (7): positive, activating epistemic emotion; sample item: curious.
- Confusion (8): negative, deactivating epistemic emotion; sample item: confused.
- Anxiety (9): negative, activating epistemic emotion; sample item: anxious.
- Frustration (10): negative, deactivating epistemic emotion; sample item: frustrated.
- Enjoyment (11): positive, activating epistemic emotion; sample item: excited.
- Boredom (12): negative, deactivating epistemic emotion; sample item: bored.

A.3 Achievement goals

The framework applied in this study is based on the common framework that distinguishes a valence dimension of goals, the approach-avoidance distinction, and a definition dimension of goals. Where that definition dimension is often operationalized as a mastery–performance distinction [26], we follow two

contemporary developments: that of distinguishing two separate evaluation standards in the mastery definition, focus on the attainment of task-based as well as self-based competence, whereas the performance goal is identified with the attainment of other-based competence [46], and the addition of the dimension of future potentials [47]. That results in the following eight scales of the AGQ (Achievement Goals Questionnaire), each scale counting three items:

- Task-approach (TAP, 13) goals: focus on the attainment of task-based competence; sample item: To get a lot of questions right on the exams in this class.
- Task-avoidance (TAV, 14) goals: focus on the avoidance of task-based incompetence; sample item: To avoid incorrect answers on the exams in this class.
- Self-approach (SAP, 15) goals: focus on the attainment of self-based competence; sample item: To perform better on the exams in this class than I have done in the past on these types of exams.
- Self-avoidance (SAV, 16) goals: focus on the avoidance of self-based incompetence; sample item: To avoid doing worse on the exams in this class than I normally do on these types of exams.
- Other-approach (OAP, 17) goals: focus on the attainment of other-based competence; sample item: To do well compared to others in the class on the exams.
- Other-avoidance (OAV, 18) goals: focus on the avoidance of other-based incompetence; sample item: To avoid doing poorly in comparison to others on the exams in this class.
- Potential-approach (PAP, 19) goals: focus on the attainment of potential-based competence; sample item: To do as well as I can possibly do on the exams in this class.
- Potential-avoidance (PAV, 20) goals: focus on the avoidance of potential -based incompetence; sample item: To avoid doing worse than my very best on the exams in this class.

A.4 Motivation and engagement

The 'Motivation and Engagement Wheel’ framework [20] describes behaviours and thoughts or cognitions that play a role in learning. Both are subdivided into adaptive and maladaptive (or obstructive) forms. The MES (Motivation and Engagement Scale) is a self-report survey based on this framework consisting of the following scales:

- Self-Belief (SB, 21): adaptive cognition; sample item: If I try hard, I believe I can do my university work well.
- Value of School (VS, 22): adaptive cognition; sample item: I a able to use some of the things I learn at university in other parts of my life.
- Learning Focus (LF, 23): adaptive cognition; sample item: I feel very pleased with myself when I really understand what I am taught at university.
- Planning (PL, 24): adaptive behaviour; sample item: I get it clear in my head what I am going to do when I sit down to study.
- Study Management (SM, 25): adaptive behaviour; sample item: When I study, I usually study in places where I can concentrate.
- Persistence (PS, 26): adaptive behaviour; sample item: If I can’t understand my university work at first, I keep going over it until I do.
- Anxiety (AN, 27): maladaptive cognition; sample item: When exams and assignments are coming up, I worry a lot.
- Failure Avoidance (FA, 28): maladaptive cognition; sample item: Often the main reason I work at university is because I don’t want people to think that I am dumb.
- Uncertain Control (UC, 29): maladaptive cognition; sample item: When I don’t do so well at university I am often unsure how to avoid that happening again.
- Self-sabotage (SS, 30): maladaptive behaviour; sample item: Sometimes I don’t try hard at assignments so I have an excuse if I don’t do so well.
- Disengagement (DS, 31): maladaptive behaviour; sample item: Each week I am trying less and less.

As a result, the four quadrants are adaptive behaviour or positive engagement and adaptive cognitions or positive motivation (the ‘boosters’), mal-adaptive behaviour or negative engagement (the ‘guzzlers’) and obstructive cognitions or negative motivation (the ‘mufflers’). From a later study [48],

- Academic Buoyancy (32); sample item: I'm good at dealing with setbacks (e.g., bad mark, negative feedback on my work).

has been added as a further positive engagement. All scales count four items.

A.5 Attitudes towards learning

Attitudes towards learning of mathematics and statistics were assessed with the SATS instrument [33], based on the expectancy-value theory [19]. The instrument contains six quantitative methods-related attitudes:

- Affect (33): students’ feelings concerning mathematics and statistics (six items); sample item: I enjoy taking QM courses.
- CognComp (34): students’ self-perceptions of their intellectual knowledge and skills when applied to mathematics and statistics (six items); sample item: I find it difficult to understand mathematical and statistical concepts (reversed).
- Value (35): students’ attitudes about the usefulness, relevance, and worth of mathematics and statistics in their personal and professional life (nine items); sample item: Mathematical and statistical skills will make me more employable.
- NoDifficulty (36): students’ perceptions that mathematics and statistics as subjects are not difficult to learn (seven items); sample item: Mathematical and statistical formulas are easy to understand.
- Interest (37): students’ level of individual interest in learning mathematics and statistics (four items); sample item: I am interested in using mathematics and statistics.

- Effort (38): the amount of work students are willing to undertake to learn the subjects (four items); sample item: I plan to study hard for every QM test.

A.6 Students’ approaches to learning

Individual approaches to cognitive learning processing strategies and metacognitive learning regulation strategies are based on Vermunt’s [21] ILS instrument. Processing strategies can be ordered from surface to deep learning approaches:

- Memorising and rehearsing (MEMO, 39, five items); sample item: I repeat the most important parts of the material until I know them by heart.
- Analysing (ANAL, 40, six items); sample item: I work through a chapter in a textbook point by point and study each finished section separately.
- Relating and structuring (REL, 41, seven items); sample item: I try to pull together everything that has been dealt with separately in a course to form a whole.
- Critical processing (CRIT, 42, five items); sample item: I compare my view of a topic in the course with the view of the authors of the book being dealt with.
- Concrete processing (CONC, 43, five items) acts as a separate category, not being part of the ordering from surface to deep approaches; sample item: I use what I learn during my activities in a course outside of my studies.

Regulation strategies are decomposed into four subscales describing the continuum of self and external regulation. The fifth scale is again not part of that continuum but describes the absence of learning regulation.

- Self-regulation of learning processes and results (SRLP, 44, six items); sample item: In order to test my progress in learning, I try, after studying the textbook, to formulate the main points in my own words.
- Self-regulation of learning content (SRLC, 45, four items); sample item: In addition to the textbook, I also study other books/articles, which relate to the content of the course.
- External regulation of learning processes (ERLP, 46, six items); sample item: If a textbook contains questions or assignments, I work them out completely as soon as I come across them while studying.
- External regulation of learning results (ERLC, 47, five items); sample item: I test my progress purely by answering the questions and doing the assignments and exercises in the textbook that the teacher tells us to do.
- Lack of regulation (LACK, 48, six items) indicating lack of regulation of any type; sample item: I realise that it is not clear to me what I need to remember and what I do not need to remember.

A.7 Academic motivations

The Academic Motivation Scale [27] is based upon Ryan and Deci's [49] model of intrinsic and extrinsic motivation. The AMS consists of seven scales with each four items, to which students respond according to the question stem "Why are you going to university?" There are seven subscales on the AMS, each counting four items, of which three belong to the intrinsic motivation scale:

- Intrinsic motivation to know (IMKnow, 49); sample item: Because I experience pleasure and satisfaction while learning new things.
- Intrinsic motivation to accomplish (IMAcc, 50); sample item: For the pleasure that I experience while I am surpassing myself in one of my personal accomplishments. And

- Intrinsic motivation to experience stimulation (IMStim, 51); sample item: For the intense feelings I experience when I am communicating my own ideas to others.

Three other subscales belong to the extrinsic motivation scale:

- Identified regulation (Emiden, 52); sample item: Because I think that a college education will help me better prepare for the career I have chosen.
- Introjected regulation (Emintro, 53); sample item: To prove to myself that I am capable of completing my college degree. And
- External regulation (Emext, 54); sample item: Because with only a high-school degree I would not find a high-paying job later on.

The final scale,

- A-motivation (Amo, 53) constitutes the absence of regulation, either externally directed or internally; sample item: Honestly, I don't know; I really feel that I am wasting my time in school.
