# Supplementary material for: Subjective data, objective data and the role of bias in predictive modelling: Lessons from a dispositional learning analytics application
Source: PLoS One. 2020 Jun 12;15(6):e0233977. doi: 10.1371/journal.pone.0233977 (PMC7292385; doi:10.1371/journal.pone.0233977)
Supplement: S2 Appendix — (PDF) [file pone.0233977.s002.pdf]

Appendix B: Descriptive statistics of all variables in the study.

Table B1. Overview of variables in the study, the means, medians and their extreme response and overconfidence scores.

Variable name	Variable acronym	Variable number in figure	Instrument	Mean	Median	ERSpos	ERSneg	ΔConfi-dence
LEnjoyment	LJO	1	AEQ: Achievement Emotions Questionnaire	4.24	4.30	0.376	0.096	0.055
LAnxiety	LAX	2		3.96	4.00	0.238	-0.640	-0.313
LBoredom	LBO	3		2.89	2.73	-0.228	-0.407	-0.116
LHopelessness	LHL	4		3.05	2.91	0.058	-0.648	-0.270
Academic Control	ASC	5		5.18	5.25	0.043	0.515	0.204
Surprise	Surprise	6	EES: Epistemic Emotion Scales	4.06	4.00	0.232	-0.251	-0.013
Curiosity	Curiosity	7		5.23	5.33	0.384	0.056	0.069
Confusion	Confusion	8		4.23	4.33	0.129	-0.511	-0.271
Anxiety	Anxiety	9		4.40	4.67	0.249	-0.558	-0.336
Frustration	Frustration	10		3.79	3.67	0.063	-0.493	-0.271
Enjoyment	Enjoyment	11		4.34	4.33	0.241	0.093	0.080
Boredom	Boredom	12		3.28	3.33	-0.175	-0.311	-0.083
Task-approach	TAP	13	AGQ: Achievement	6.27	6.33	0.433	0.130	0.080
Task-avoidance	TAV	14		6.17	6.33	0.412	-0.003	0.054

Self-approach	SAP	15	Goals Questionnaire	5.77	6.00	0.514	-0.047	-0.025
Self-avoidance	SAV	16		5.77	6.00	0.505	-0.081	-0.024
Other-approach	OAP	17		4.28	4.00	0.345	-0.081	0.076
Other-avoidance	OAV	18		4.74	5.00	0.372	-0.142	0.013
Potential-approach	PAP	19		6.48	6.67	0.512	0.033	-0.029
Potential-avoidance	PAV	20		5.87	6.00	0.492	-0.068	0.012
Self-belief	SB	21	MES: Motivation and Engagement Scale	5.98	6.00	0.364	0.266	0.165
Value of School	VS	22		6.03	6.00	0.479	0.079	0.059
Learning Focus	LF	23		6.34	6.50	0.521	0.054	-0.053
Planning	PL	24		4.84	5.00	0.385	0.047	0.076
Task Management	SM	25		5.62	5.75	0.370	0.050	0.069
Persistence	PS	26		5.58	5.75	0.439	0.169	0.073
Anxiety	AN	27		4.63	4.75	0.302	-0.434	-0.316
Failure Avoidance	FA	28		2.49	2.25	0.117	-0.376	-0.187
Uncertain Control	UC	29		3.42	3.50	0.030	-0.455	-0.257
Self-Sabotage	SS	30		2.18	2.00	-0.140	-0.322	-0.116
Disengagement	DS	31		1.73	1.50	-0.224	-0.282	-0.094
Academic Buoyancy	AB	32		4.20	4.25	-0.092	0.308	0.255
Affect	Affect	33		4.32	4.33	-0.063	0.542	0.476

Cognitive Competence	CognComp	34	SATS: Survey of Attitudes Toward Statistics	4.90	5.00	-0.002	0.508	0.497
Value	Value	35		5.28	5.33	0.259	0.324	0.231
NoDifficulty	NoDifficulty	36		3.14	3.14	-0.166	0.238	0.137
Interest	Interest	37		5.27	5.50	0.384	0.090	0.025
Effort	Effort	38		6.56	6.75	0.442	0.030	0.030
Memorising	MEMO	39	ILS: Inventory of Learning Styles, processing	4.45	4.60	0.402	-0.128	-0.008
Analysing	ANAL	40		4.74	4.67	0.512	0.022	0.082
Relating	REL	41		5.04	5.00	0.469	0.052	0.093
Critical processing	CRIT	42		4.62	4.75	0.338	-0.011	0.073
Concrete processing	CONC	43		4.89	5.00	0.461	-0.020	0.061
Self-regulation Process	SRLP	44	ILS: Inventory of Learning Styles, regulation	4.70	4.71	0.485	-0.085	0.021
Self- regulation Content	SRLC	45		4.08	4.00	0.369	-0.094	-0.011
External- regulation Process	ERLP	46		4.63	4.67	0.428	-0.068	0.016
External- regulation Results	ERLC	47		5.11	5.20	0.485	0.034	0.046
Lack of regulation	LACK	48		3.96	4.00	0.149	-0.519	-0.256
Intrinsic Motivation, to know	IMKnow	49	AMS: Academic Motivation Scales	5.43	5.50	0.516	-0.049	0.058
Intrinsic Motivation, accomplish	IMAcc	50		4.87	5.00	0.562	-0.159	0.033
Intrinsic Motivation, stimulation	IMStim	51		4.11	4.25	0.412	-0.214	0.020
Identified regulation	Emiden	52		5.97	6.00	0.433	-0.020	0.017

Introjected regulation	Emintro	53		4.85	5.00	0.466	-0.278	-0.043
External regulation	Emext	54		5.62	5.75	0.326	-0.078	0.007
A-motivation	Amo	55		1.52	1.25	-0.147	-0.244	-0.133
Final grade	Grade	56	Performance	5.85	6.00	-0.042	0.311	-0.008
Math exam	MathExam	57		12.3	12.0	-0.062	0.278	-0.038
Statistics exam	StatsExam	58		11.8	12.0	-0.026	0.273	0.023
Math quizzes	MathQz	59		1.50	1.58	0.037	0.237	-0.084
Statistics quizzes	StatsQz	60		1.49	1.57	-0.025	0.173	0.006
BlackBoard logs	BB	61	Traces	297	292	0.139	0.032	-0.035
Mastery Sowiso	SowisoMast	62		0.778	0.929	0.107	0.027	-0.066
Mastery MSL	MSLMast	63		0.773	0.928	0.116	0.046	-0.054
Time Sowiso	SowisoTime	64		28.3h	23.3h	0.118	-0.054	-0.036
Time MSL	MSLTime	65		25.0h	22.2h	0.105	-0.065	-0.047
Attempts Sowiso	SowisoAtt	66		745	746	0.139	-0.111	-0.024
Attempts MSL	MSLAtt	67		207	225	0.122	-0.020	-0.054
Solutions Sowiso	SowisoSol	68		325	278	0.092	-0.187	0.002
