# Supplementary material for: Subjective data, objective data and the role of bias in predictive modelling: Lessons from a dispositional learning analytics application
Source: PLoS One. 2020 Jun 12;15(6):e0233977. doi: 10.1371/journal.pone.0233977 (PMC7292385; doi:10.1371/journal.pone.0233977)
Supplement: S3 Appendix — (PDF) [file pone.0233977.s003.pdf]

Appendix C: Correlations of mean response styles measures.

Table C1. Correlations of mean response styles measures.

	ARS	ARSW	DARS	DARSW	MRS	MLRS	NARS	NARSW	RR	NCR	ERSneg1	ERSpos1	ERSneg2	ERSpos2
ARS	1.000													
ARSW	.817	1.000												
DARS	-.647	-.403	1.000											
DARSW	-.438	-.082	.874	1.000										
MRS	-.532	-.575	-.301	-.422	1.000									
MLRS	-.382	-.744	-.211	-.552	.712	1.000								
NARS	.919	.685	-.896	-.708	-.155	-.113	1.000							
NARSW	.885	.827	-.808	-.628	-.210	-.269	.935	1.000						
RR	.096	.343	.237	.447	-.383	-.548	-.067	.016	1.000					
NCR	-.043	.106	.135	.197	-.097	-.183	-.095	-.028	.543	1.000				
ERSneg1	-.114	.266	.447	.793	-.353	-.653	-.298	-.240	.516	.216	1.000			
ERSpos1	.471	.870	-.171	.147	-.400	-.722	.363	.597	.406	.200	.470	1.000		
ERSneg2	-.352	-.012	.795	.965	-.442	-.634	-.617	-.554	.455	.188	.752	.168	1.000	
ERSpos2	.741	.964	-.294	.023	-.600	-.835	.584	.740	.381	.101	.304	.809	.103	1.000

Note: correlations larger than .105 in absolute size are statistically significant at $p<.001$; correlations larger than .080 in absolute size are statistically significant at $p<.01$.
