# Supplementary material for: Subjective data, objective data and the role of bias in predictive modelling: Lessons from a dispositional learning analytics application
Source: PLoS One. 2020 Jun 12;15(6):e0233977. doi: 10.1371/journal.pone.0233977 (PMC7292385; doi:10.1371/journal.pone.0233977)
Supplement: Attachment — Submitted filename: Response to Reviewers.docx [file pone.0233977.s004.docx]

Rebuttal.

| **Reviewers’ comments** | **Authors’ response** |
| --- | --- |
| Thank you for submitting your manuscript to PLOS ONE. After careful consideration, we feel that it has merit but does not fully meet PLOS ONE’s publication criteria as it currently stands. Therefore, we invite you to submit a revised version of the manuscript that addresses the points raised during the review process. | Dear academic editor, dear Vitomir,  We appreciate the feedback and recommendations we received from you and the two reviewers, specifically those from Fernandez. We have addressed all of them in the revised version of the paper and elaborate them in this rebuttal. |
| 2. We note that you have stated that you will provide repository information for your data at acceptance. Should your manuscript be accepted for publication, we will hold it until you provide the relevant accession numbers or DOIs necessary to access your data. | The data, the MPlus and SPSS codes, and the main components of the output are archived in DANS, the Data Archiving and Networked Services of the NOW, the Dutch organization of scientific research. DANS is an open access resource. The draft version of this archive, labelled Tempelaar, D, 2020, "Replication Data for PlosOne 2020 manuscript Tempelaar ea", has received the unique handle: https://hdl.handle.net/10411/YAF7CJ, DataverseNL, DRAFT VERSION  Upon receiving the DOI of the publication, the archive can be finalized. |
| **Reviewer 1** |  |
| 1. The authors mention seven-point Likert scales. Was each of the points in the scale paired with a label or only the anchors of the scale were labelled? This is essential as the former case requires multinomial (polytomous) logistic regression whereas the latter can be analysed via (linear) regression. | We are very grateful for the detailed feedback from Reviewer 1. Our research applies eight different self-report instruments, as reported in Table 1. These eight instruments were designed with different types of Likert response scales: different length and different labels. To simplify and ease the responses to these instruments, we applied the same length for all instruments, and indeed fixed the labels to include the three anchors only: the negative pole, the neutral anchor and the positive pole. This explanation is added. |
| 2. Data analysis section: the author mention that different statistical options were done but there is no justification to do so. I recommend considering labelling it ‘multiverse analyses’. Arguments for this approach are given in Steegen et al (2016). | We are new to this development of ‘multiverse analysis’ but can see the merits of this approach. We agree that our study would have been a perfect situation to apply this new approach. We have included a reference to Steegen’s work (she is nearly my neighbor, being associated to Leuven University) in the Data analysis section, and a further reference in the ‘future directions’ section since this suggestion is indeed worthwhile to adopt in future studies (it is too wide-ranging to adopt it in the current study). We hope that the main goal of multiverse analysis, the validation of empirical research outcomes by using other statistical methods or other versions of data sets, is opened by other researchers applying response styles. |
| 3. N-points Likert scales have been in use since 1932 when Rensis Likert proposed them. But there’s been progress as to their use. One approach is that the more points in the scale, the larger the control of Type I errors (see Sangthong, 2020). A better approach would be to make use of slider rating scales in that it produces bounded and continuous distributions (e.g. values between 1 and 7 but that can take, say, two decimal places). These distributions enable more fined grained results. These issues need to me considered in the discussion. | The Sangthong (2020) paper was new for us, but we were happy to see that his main conclusions, use long Likert scales, 7-point or 10-point, and use sample sizes of at least 100, are satisfied in our research. We have added this reference and a short discussion of the role of the length of the scale and the sample size to the data analysis section. We have added the reference to the slider rating scales to the section of future research, as part of the multiverse analysis. |
| 4. The authors speak of ‘endogeneity’ (largely used in econometrics and understood as an independent variable being correlated with the error term); is this concept seems to be made analogous to ‘meditation analysis’? definition of both and their distinction needs to be made explicit via a footnote. | Yes, that is correct. Since PlosOne forbids the use of footnotes, we have included the following explanation, within brackets, in the text: (the external validation of our extreme response measures, by investigating their role in the explanation of responses to an instrument not included in the calculation of extreme response measures). |
| 5. Results section, table 2: the authors use alpha coefficients. I suggest reading McNeish (2018) and reconsider using another coefficient. | We have read the McNeish article: another new area for us. We decided to address this comment by adding the Omega measure to Table 2, which now contains both alpha and omega measures. The reason to complement with omega, rather than substitute by omega, is that researchers in the domain of empirical educational studies are an important audience for us. These people, like us, are raised in the world of APA style formats, that require the description of alpha measures when using survey data. Replacing the alpha measures by omega measures, certainly when such measures are always ‘more positive’, will be a suspicious act in the eyes of such researchers, and we should try to avoid that impression. Therefore, we have added the Omega measures to Table 2, and have included in the main text references to the McNeish article and the Bandalos book from which we took the MPlus code to calculate the Omage measures.  By the way: except for the two ERSneg measures, all other variables have Omega measures that are essentially equal to the alpha measures. |
| 6. Table 2: distributions appear to be non-normal (based on the skewness estimations); in non-normal cases, the mean isn’t the most unbiased estimator of location. A simpler and robust alternative is the median or 20% trimmed mean. | We have added median scores to Table 1 (now Appendix B) and Table 2 (now Table 1). Probably due to the large sample size, differences between median scores and mean scores are quite small (except for some of the trace measures, contained in Appendix B). |
| 7. The authors performed several analyses and reported them via several tables. I wonder if there is a way those tables can be sent to a supplementary material so the main message is given in the text. Perhaps an alternative is to summarise those tables in a few graphs. | Yes, we agree and already when giving shape to the first version, we have experimented with alternative displays of the information contained in the tables, including graphics. But with each attempt, we judged it to be less satisfying than the simple but dull tables. The problem is that all tables contain data with different ranges, so that we either arrive at graphs with large parts of white space, or we need to do nasty transforms of the data to get all of them on a comparable range. The other problem is that all Tables 3-20 contain similar data consisting of regression betas and R^2^ values, so there is no good reason the turn some into graphs leaving the others as tables.  In the end, we decided to place Table 1 in a statistical appendix: it is by far the largest table, spanning two pages, and it is the least important table for understanding the main outcomes of our study. |
| 8. Conclusions section: the last sentence was incomplete. | Sentence has been completed. |
| Extra comments: the authors used ordinary linear regression (or OLS). Most likely data don’t meet assumptions needed for those models to perform well. If there is true interest in estimating unbiased beta weights, I recommend using robust linear regression methods (see for example Wilcox, 2017). | Thank you for this suggestion. A robust regression is an appropriate alternative to OLS in situations where outliers can heavily bias the beta estimation. However, given the large sample (N=1080), and relatively small skewness values, OLS can produce reasonable estimates in this case.  We have noted robust regression as a potential direction for future research in the discussion. |
| **Reviewer 2** |  |
| 1. One slight issue on the second page of the main article - it is stated that "In the educational psychology literature, it is widely acknowledged that although questionnaires…", but the two references given at the end of the sentence are based in the marketing domain. | We are also extremely grateful to the feedback from Reviewer 2. You are right: we should not reference here to educational research, but to research in the survey method domain. Revised in that manner. |
| 2. In Table 1 - it appeared that the variable name and variable acronyms have been swapped between columns in the rows from MEMO down - is that right? | Right again. Part of the 1^st^ and 2^nd^ columns of Table 1, now Appendix B, got mixed up. We corrected this. |
| 3. The paper contains a few grammatical errors and missing words - so would benefit from a thorough proofread prior to publication. | Yes, the revised paper was proofread by two readers. |
